# Supplementary material for: Nucleosome Remodeling at the Yeast PHO8 and PHO84 Promoters without the Putatively Essential SWI/SNF Remodeler
Source: Int J Mol Sci. 2023 Mar 3;24(5):4949. doi: 10.3390/ijms24054949 (PMC10003099; doi:10.3390/ijms24054949)

Supplementary Figures S1-S7 for Lieleg C et al.

„Nucleosome remodeling at the yeast *PHO8* and *PHO84* promoters without the putatively essential SWI/SNF remodeler”

In the following, original scan images of the films that monitored the blots shown for each indicated main figure are shown underneath each main figure panel so that the corresponding lanes are vertically aligned. If several films with different exposure times were used for the same blot, all used films are shown.

Figure S1

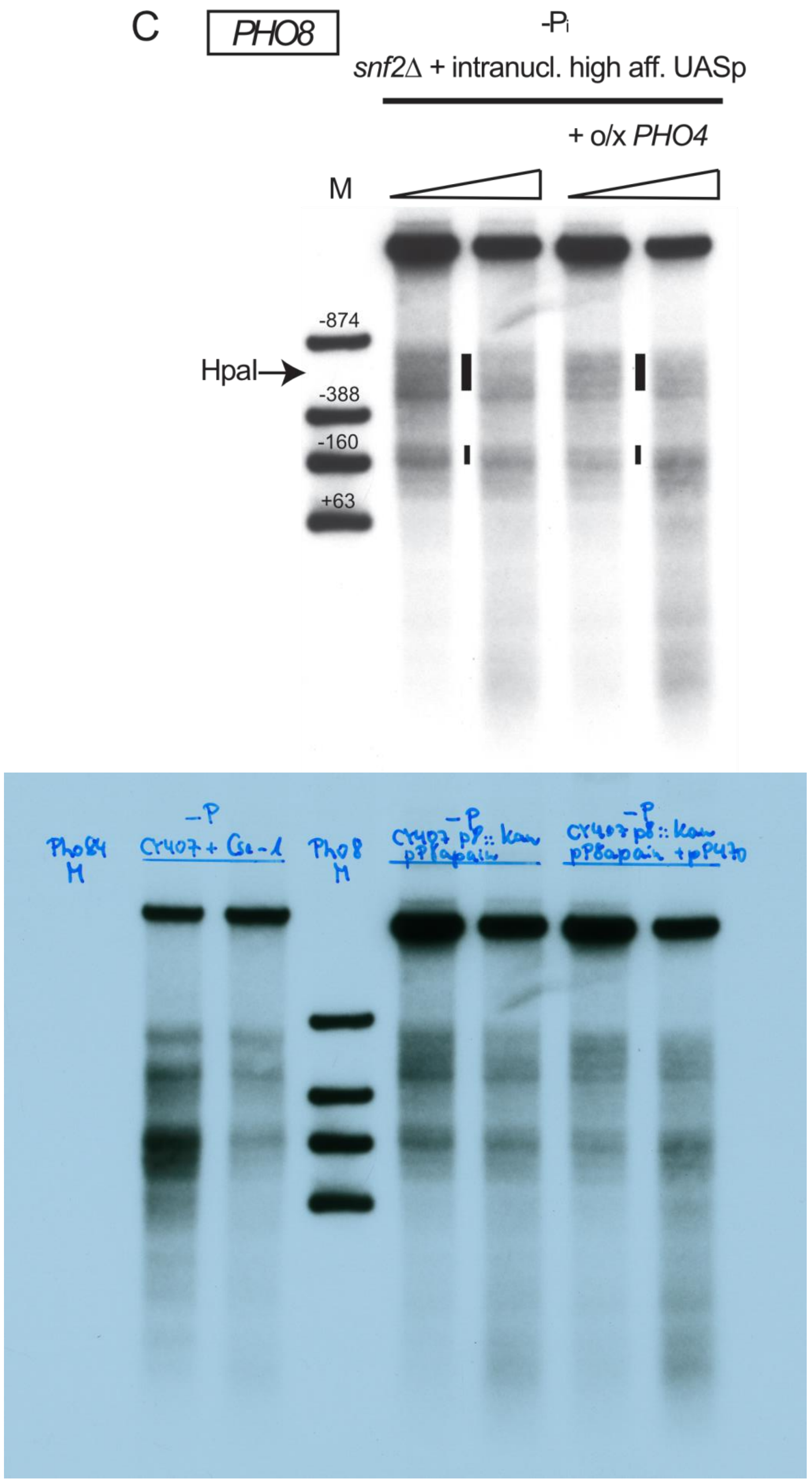

Figure S1. The main Figure 1C is shown here again on the right and the original scan image used to generate this main figure is shown below. Two lanes to the left of the marker lane that contained samples not included in the manuscript were cut away from the original blot image.

Figure S2

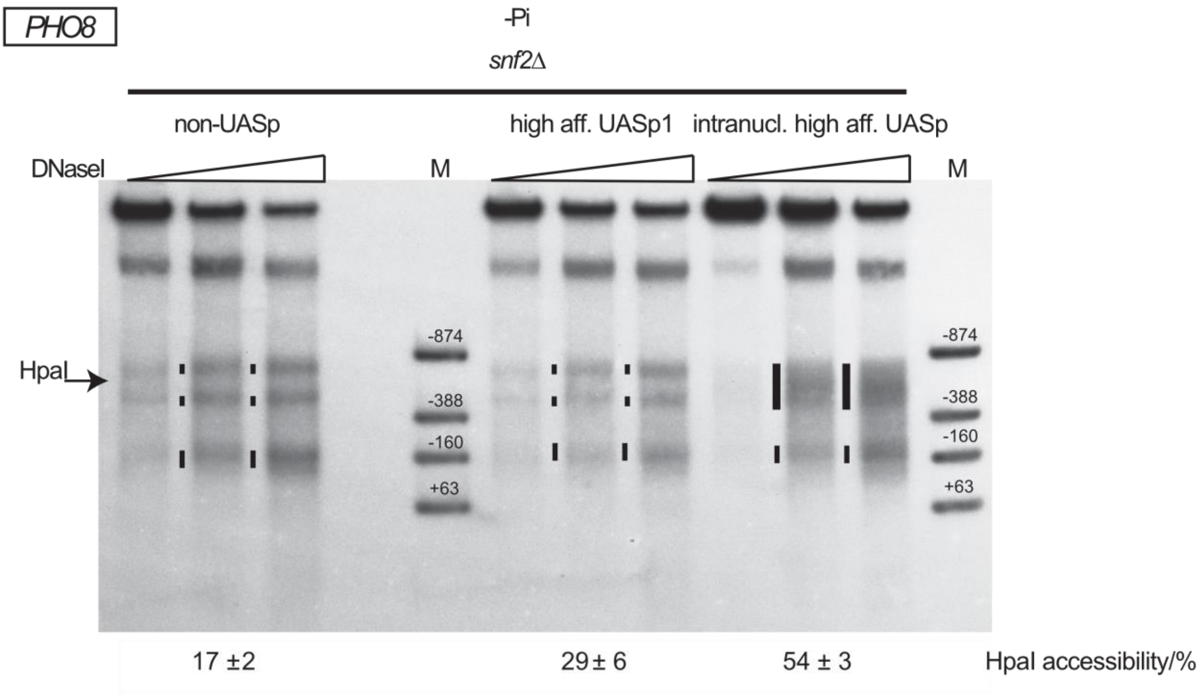

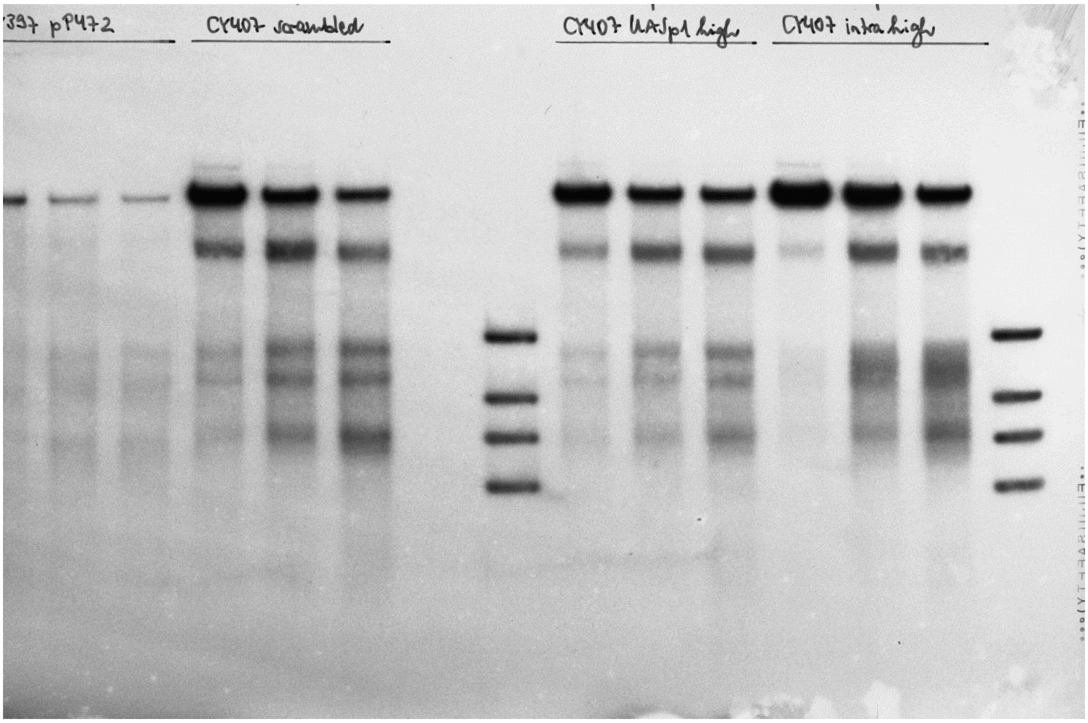

Figure S2. The main Figure 2B is shown here again on top and the original scan image used to generate this main figure is shown below. The three leftmost lanes that contained samples not included in the manuscript were cut away from the original blot image.

Figure S3

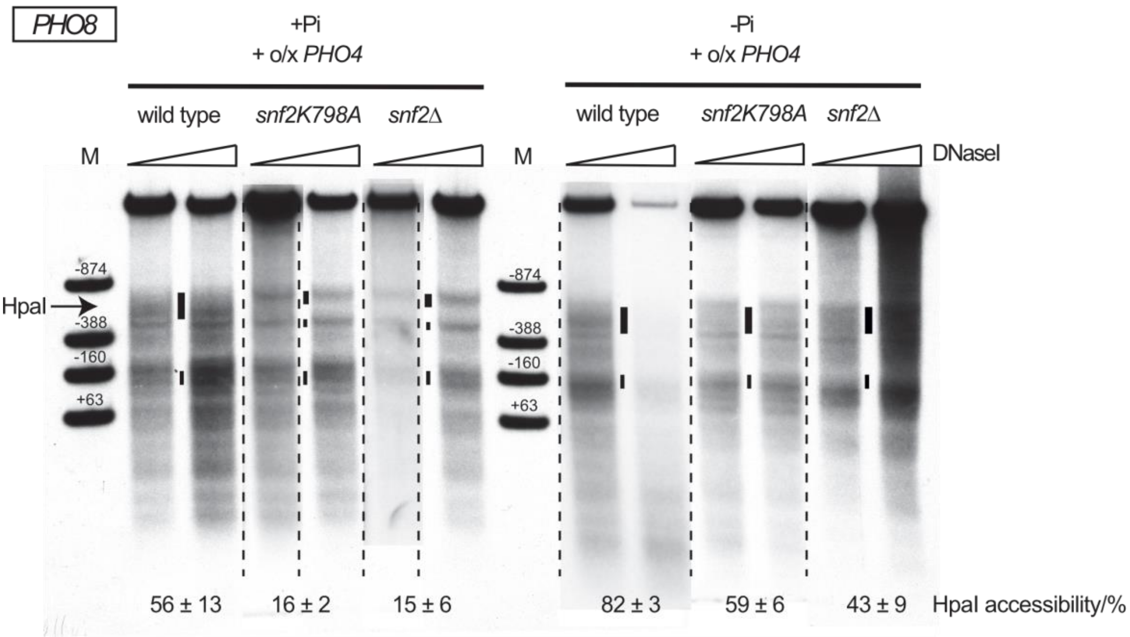

Overnight exposure

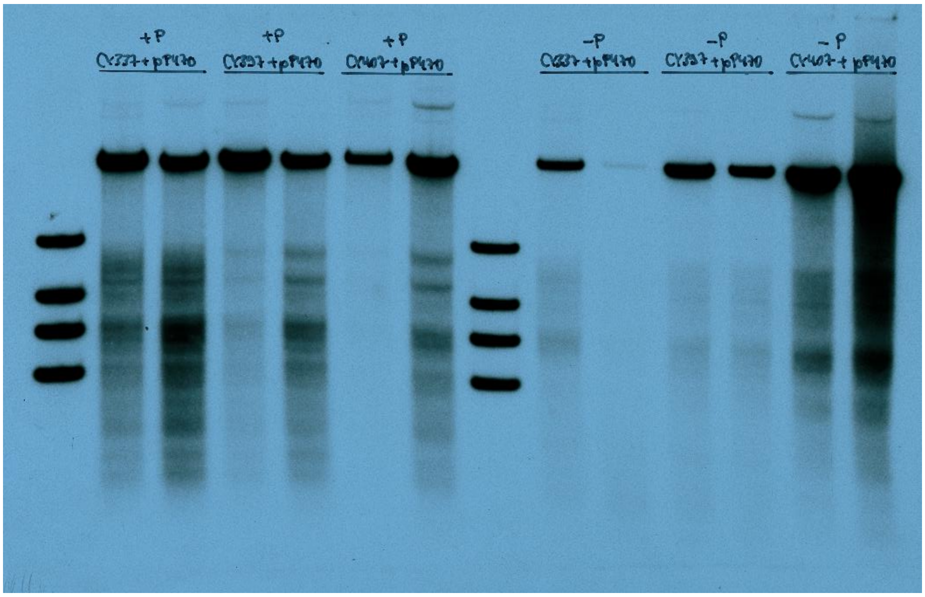

Figure S3. The main Figure 3 is shown here and on the next page again on top and the original scan images used to generate this main figure are shown below here and on the following page. The final main Figure 3 was spliced together (indicated by thin vertical dashed lines) with lane images of the three different exposure times indicated on this and the following page.

Figure S3 (contd.)

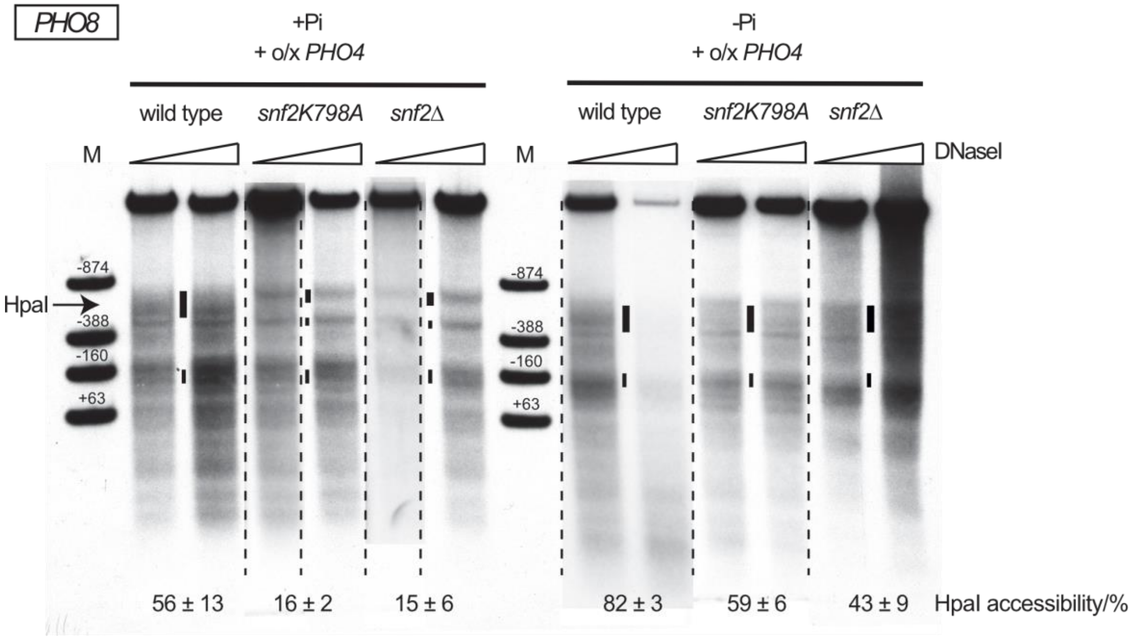

Five days exposure

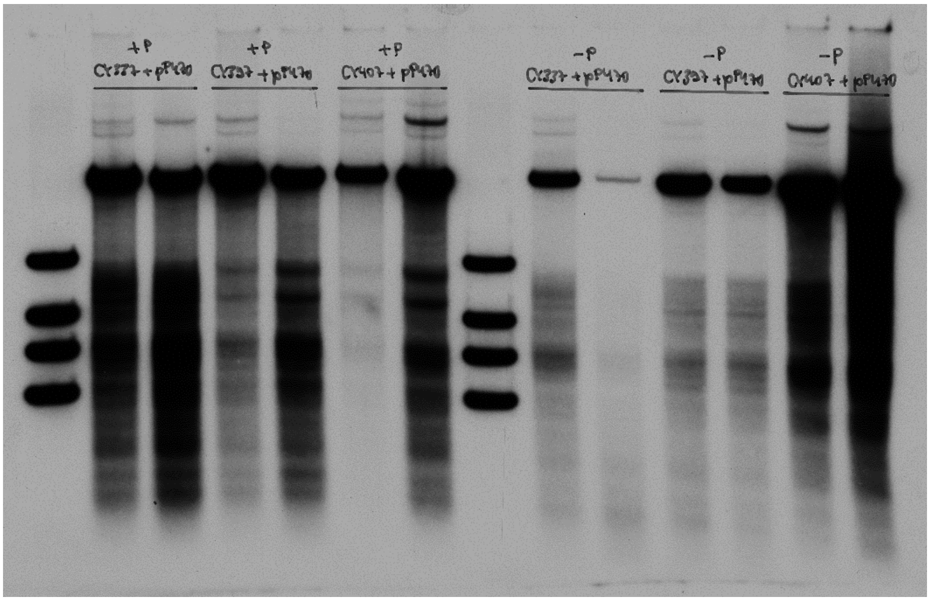

Seven days exposure

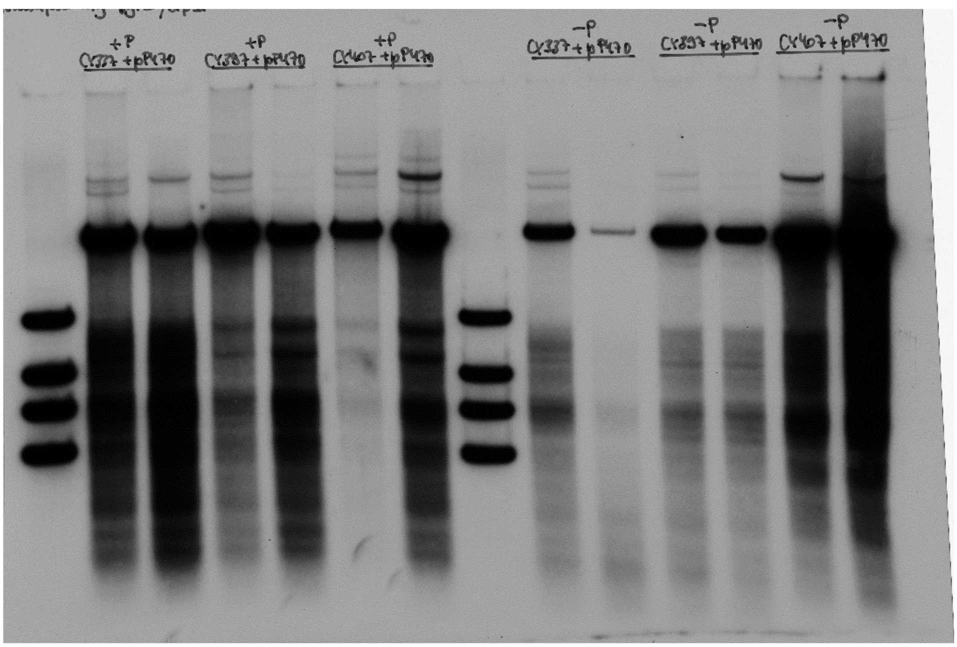

Figure S4

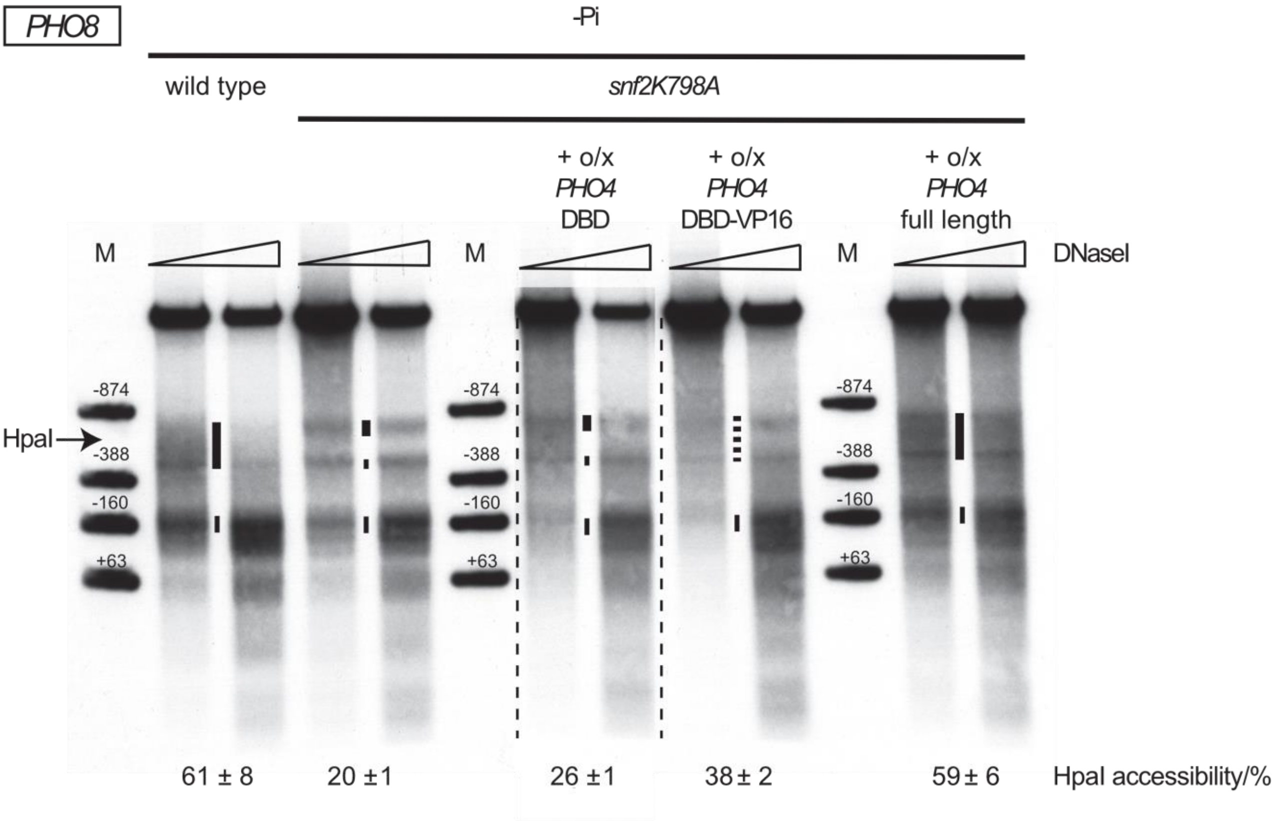

Overnight exposure

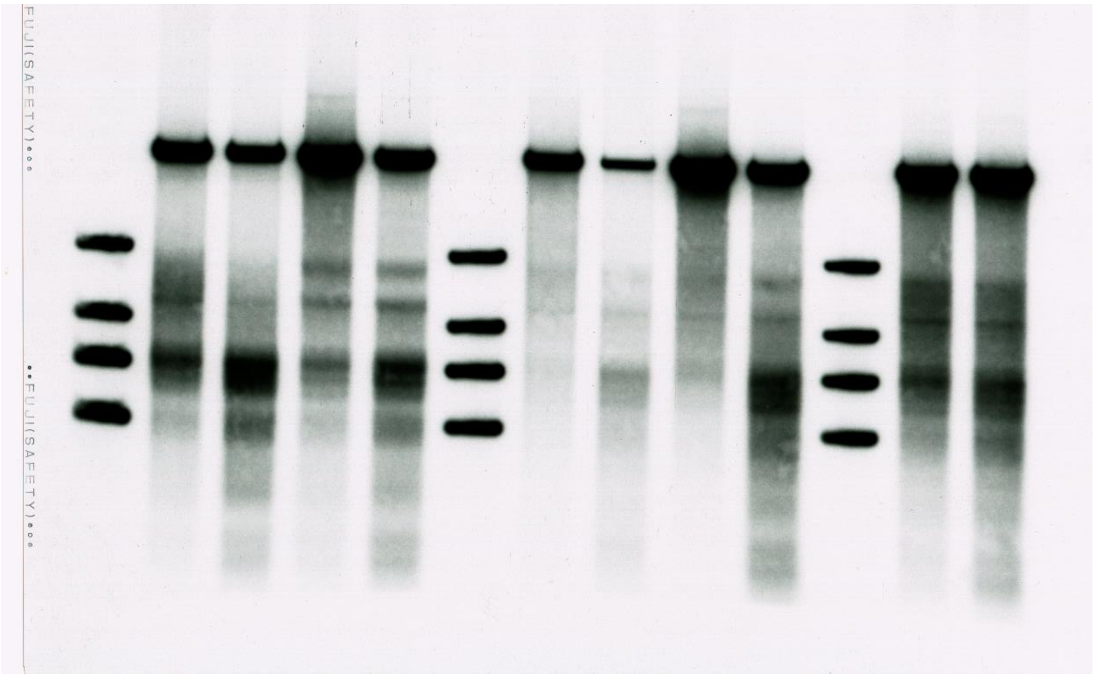

Figure S4. The main Figure 4 is shown here and on the next page again on top and the original scan images used to generate this main figure are shown below here and on the following page. The final main Figure 4 was spliced together (indicated by thin vertical dashed lines) with lane images of the two different exposure times indicated on this and the following page.

Figure S4 contd.

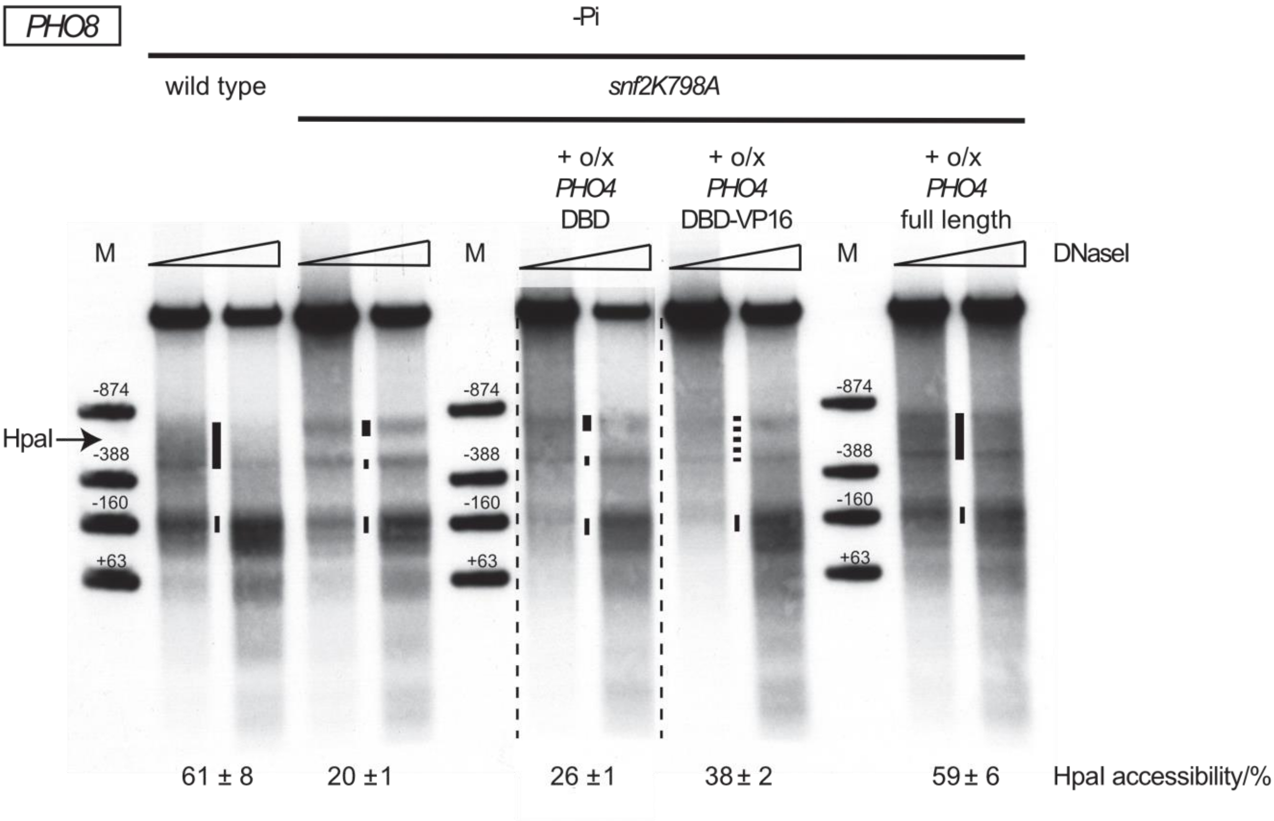

Two days exposure

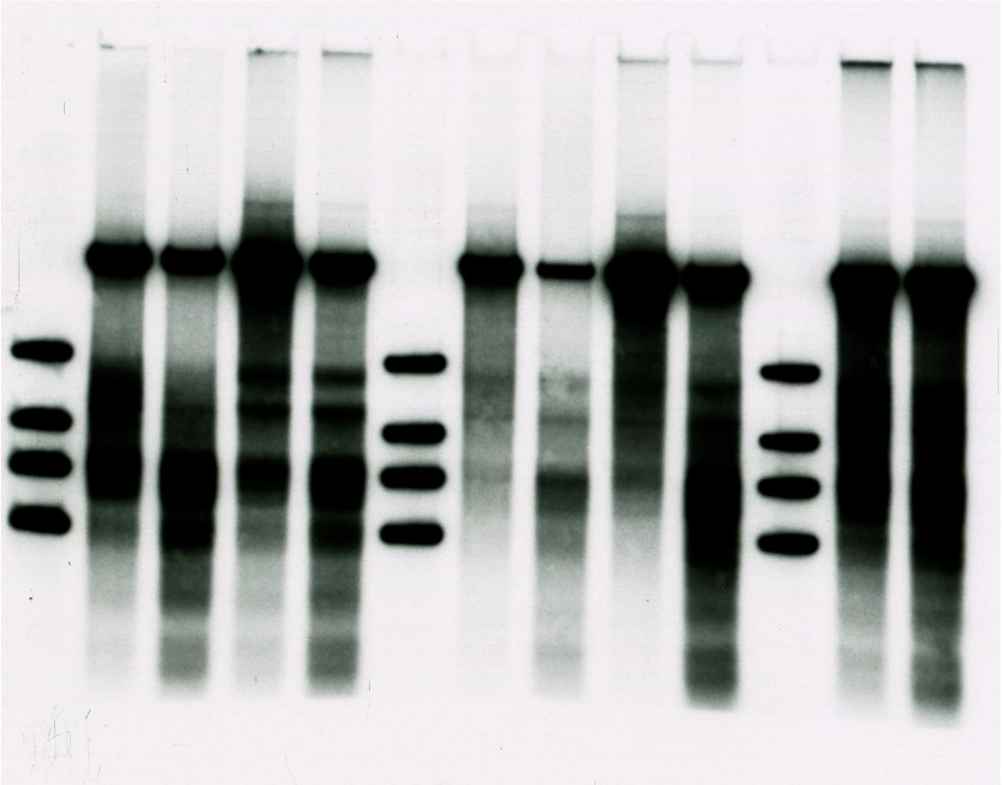

Figure S5

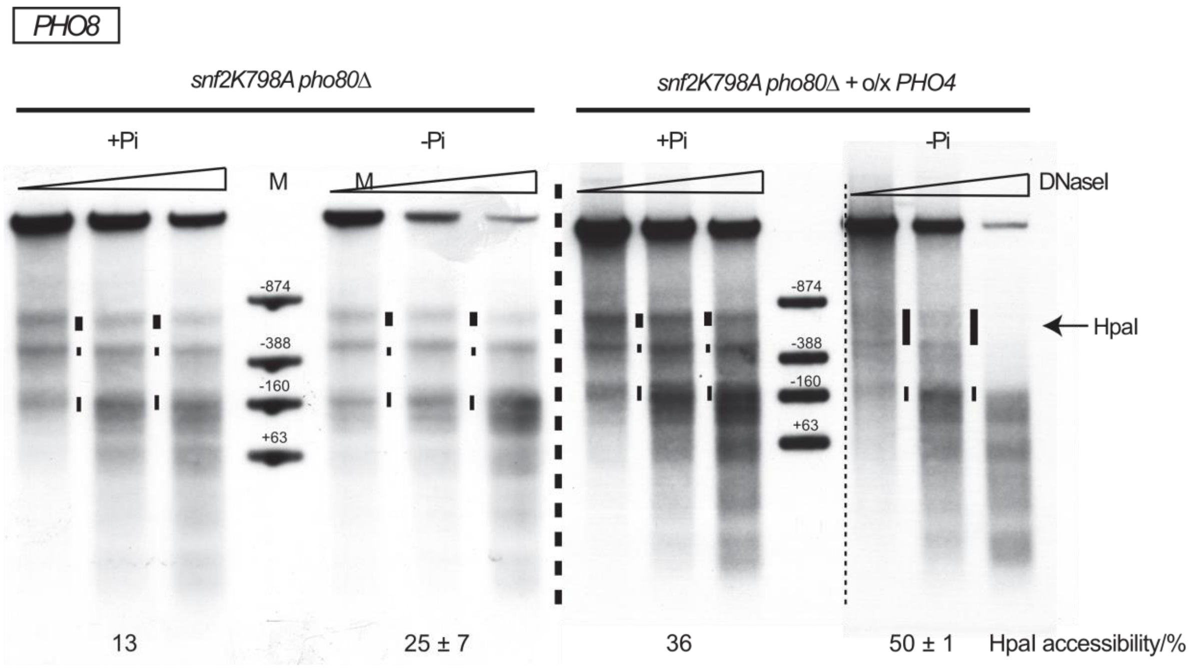

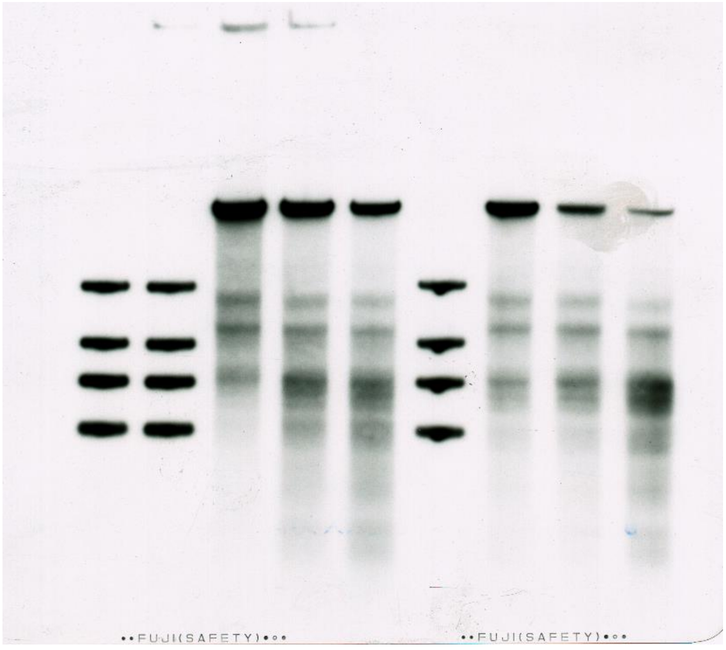

Figure S5. The main Figure 5 is shown here and on the next two pages again on top and the original scan images used to generate this main figure are shown below here and on the following two pages. The final main Figure 5 was spliced together from two different blots (separated by thick vertical dashed line) and from lane images of two different exposure times (separated by thin vertical dashed line) shown on the following two pages. For the left hand side from the thick vertical line of main Figure 5, the two leftmost marker lanes of the original blot image were cut away.

Figure S5 (contd.)

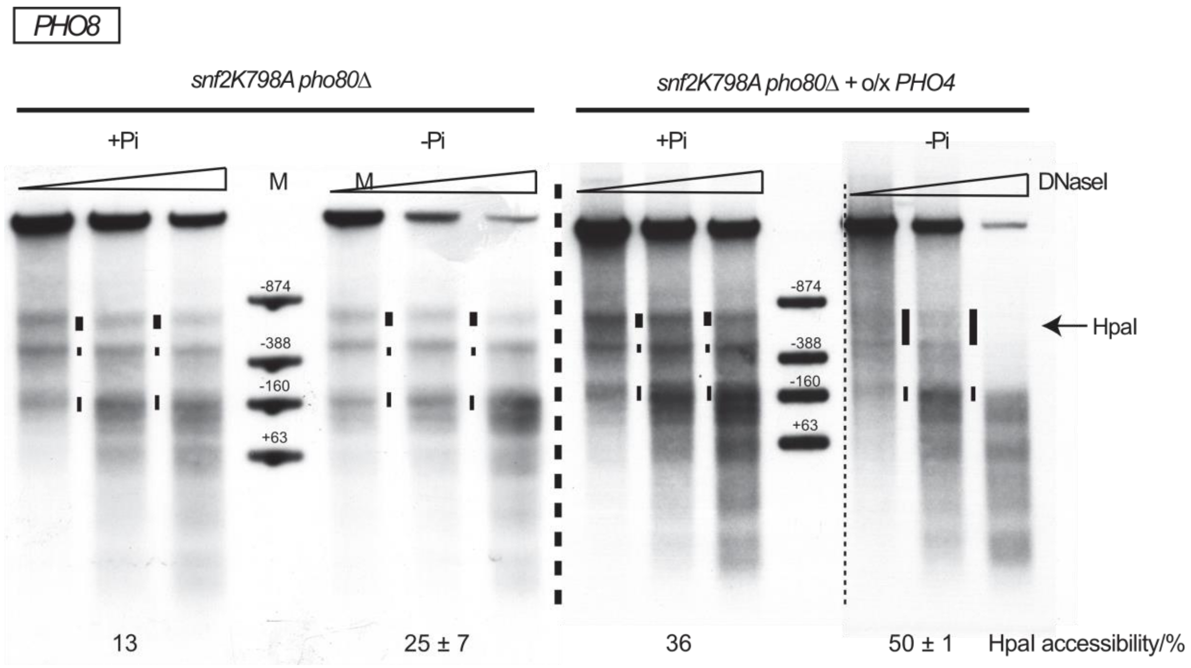

Overnight exposure

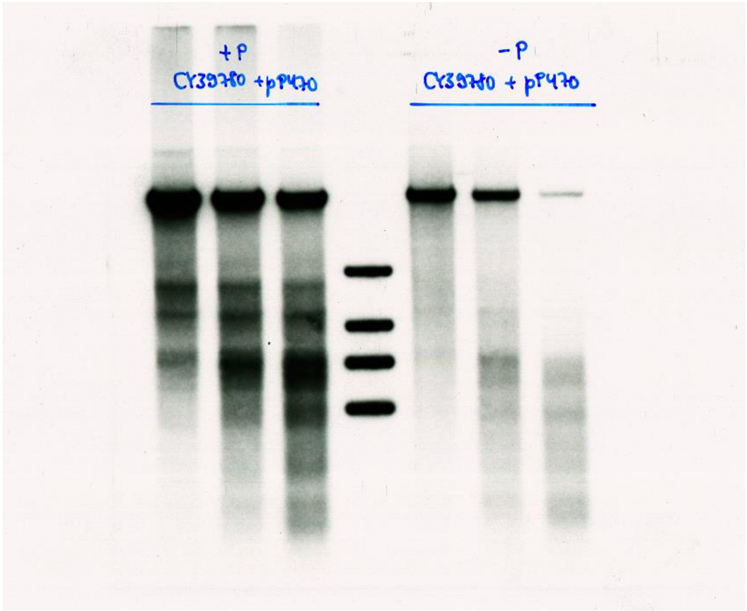

Figure S5 (contd.)

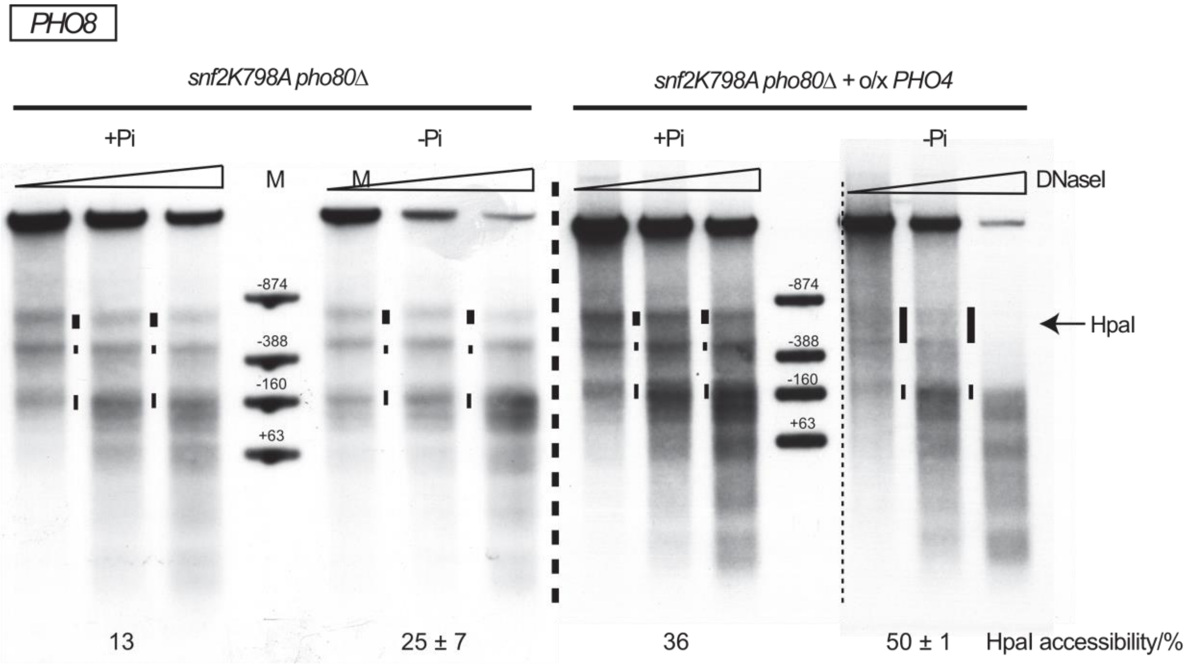

Long exposure

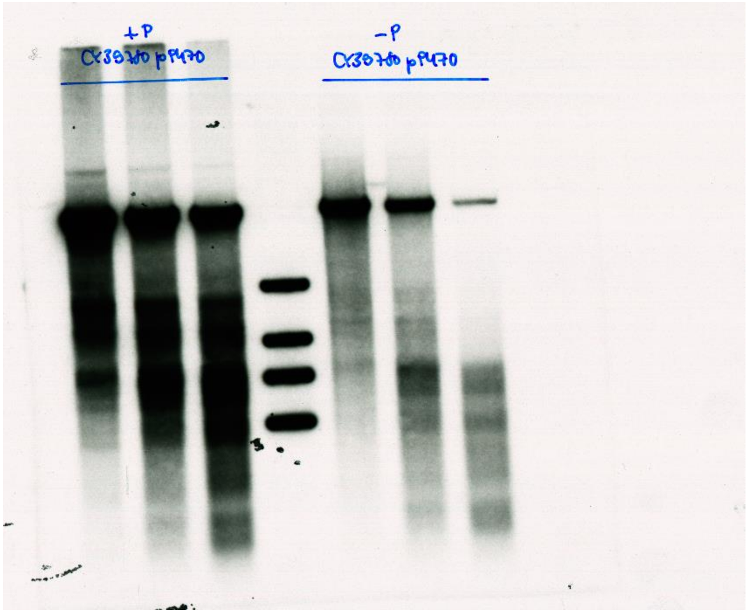

Figure S6

B

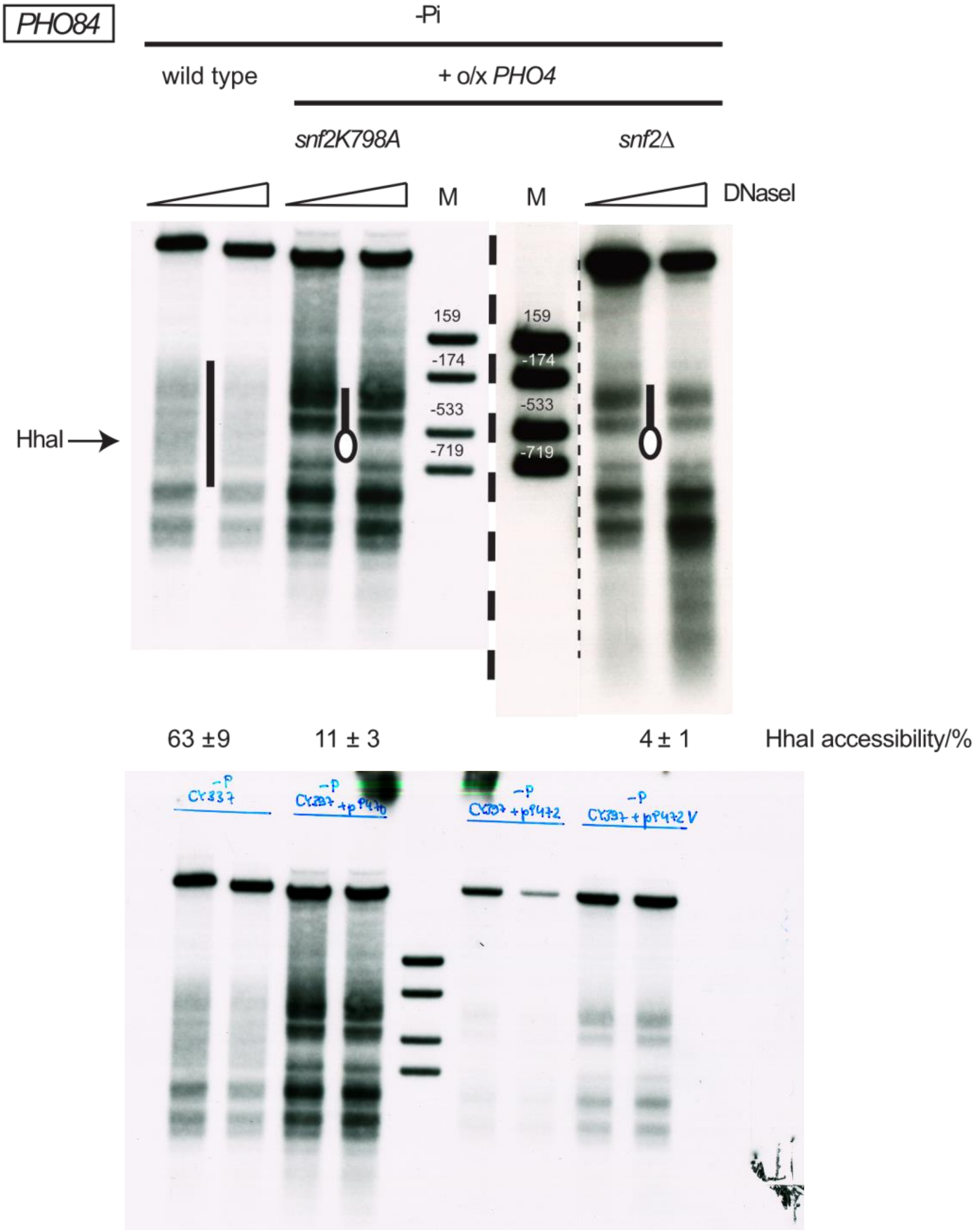

Figure S6. The main Figure 7B is shown here and on the next page again on top and the original scan images used to generate this main figure are shown below here and on the following page. The final main Figure 7B was spliced together from two different blots (separated by thick vertical dashed line) and from lanes (separated by thin vertical dashed line) that were horizontally moved together after cutting away the intervening lanes. For the left hand side from the thick vertical line of main Figure 7B, four lanes to the right of the marker lane with samples not used in the manuscript were cut away from the original blot image. For the right hand side from the thick vertical line of main Figure 7B, two rightmost lanes and the marker lane were horizontally moved together after the five intervening lanes were cut away from the origional blot image (see next page).

Figure S6 (contd.)

B

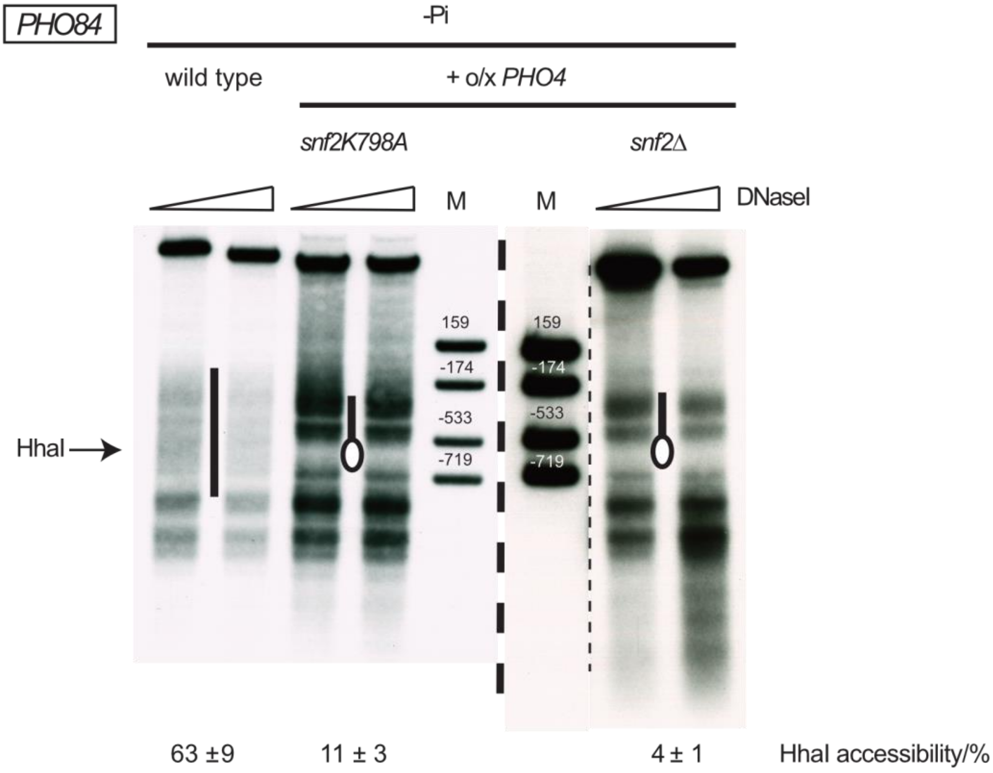

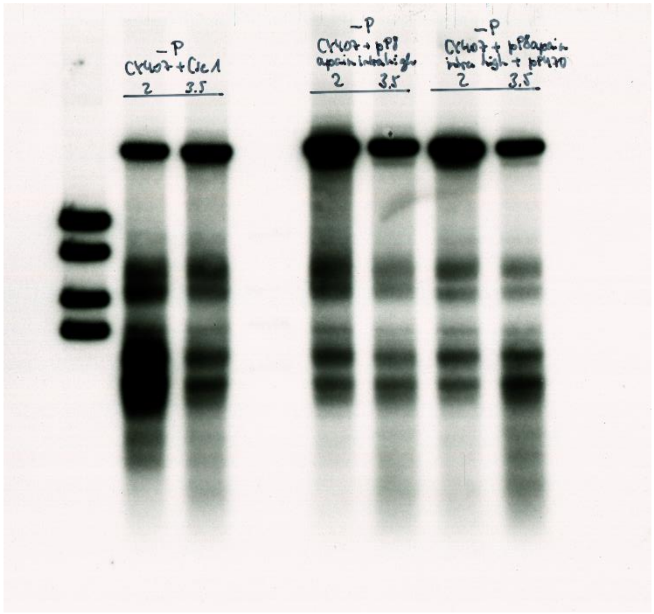

lanes cut away

Figure S7

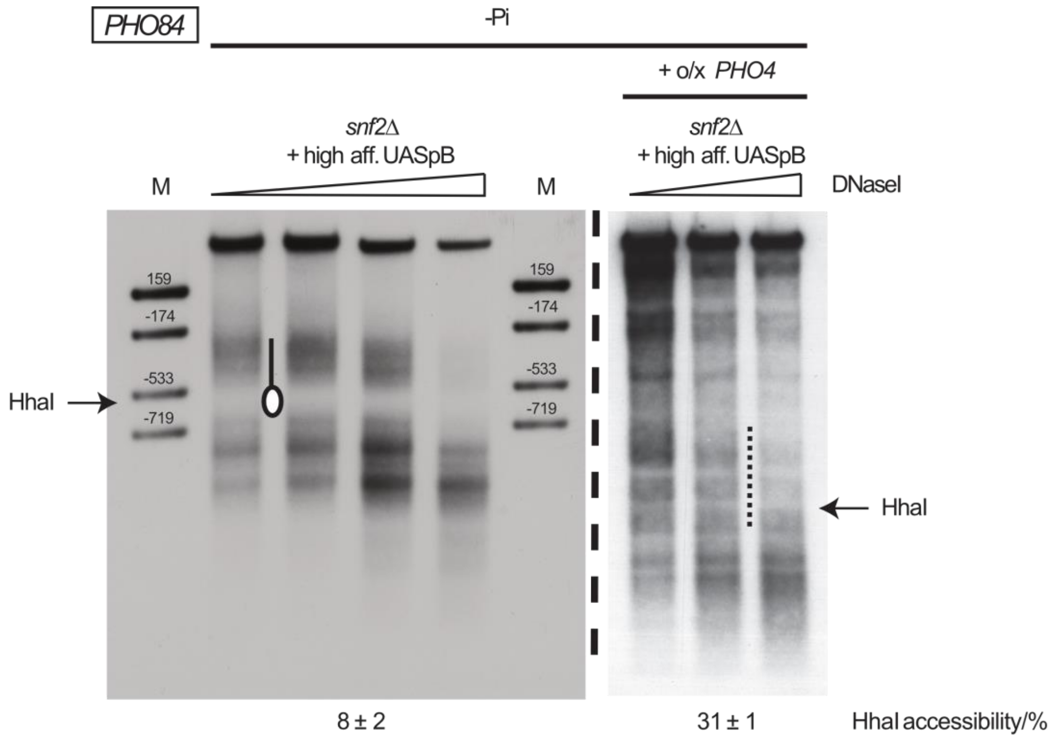

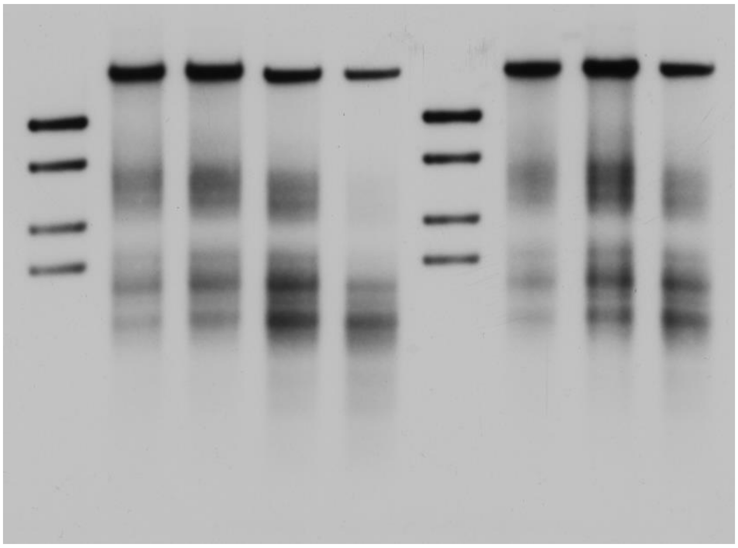

Figure S7. The main Figure 8 is shown here and on the next page again on top and the original scan images used to generate this main figure are shown below here and on the following page. The final main Figure 8 was spliced together from two different blots (separated by thick vertical dashed line) that are shown here and on the following page. For the left hand side from the thick vertical line of main Figure 8, three lanes to the right of the marker lane with samples not used in the manuscript were cut away from the original blot image. For the right hand side from the thick vertical line of main Figure 8, only the three leftmost lanes of the original blot image were used (see next page).

Figure S7 (contd.)

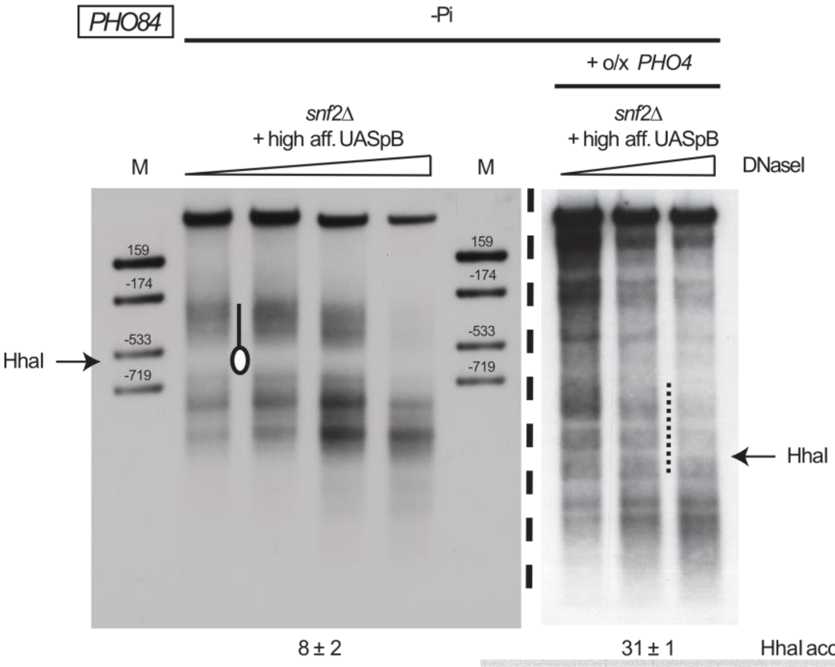

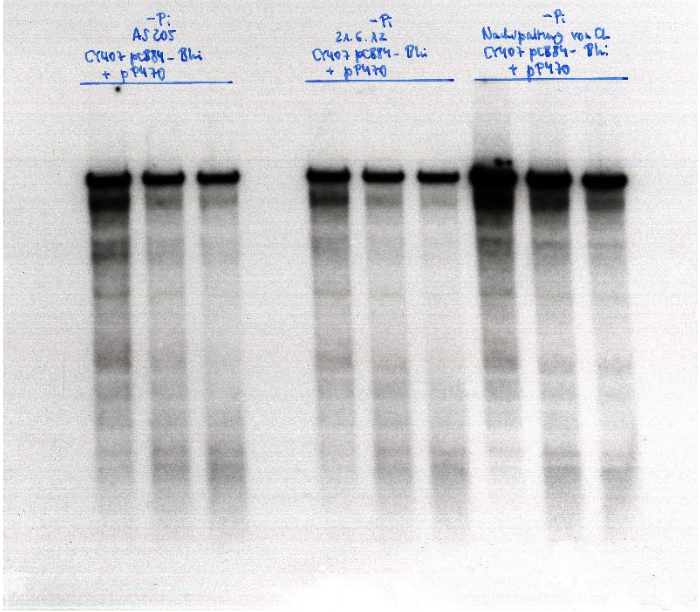

Supplement: Supplementary file 1 [file ijms-24-04949-s001.zip › ijms-2111029-Supplementary.pdf]
